# Super-resolution mapping of glutamate receptors in *C. elegans* by confocal correlated PALM

**DOI:** 10.1038/srep13532

**Published:** 2015-09-01

**Authors:** Jeroen Vangindertael, Isabel Beets, Susana Rocha, Peter Dedecker, Liliane Schoofs, Karen Vanhoorelbeeke, Johan Hofkens, Hideaki Mizuno

**Affiliations:** 1 Laboratory for Photochemistry and Spectroscopy, Division of Molecular Imaging and Photonics, Department of Chemistry, KU Leuven. Celestijnenlaan 200F, 3001 Heverlee, Belgium; 2 Laboratory for Functional Genomics and Proteomics, Division of Animal Physiology and Neurobiology, Department of Biology, KU Leuven. Naamsestraat 59, 3000 Leuven, Belgium; 3 Laboratory for Thrombosis Research, Interdisciplinary Research Facility Life Sciences, KU Leuven Kulak. E. Sabbelaan 53, 8500 Kortrijk, Belgium; 4 Nano-Science Center, Department of Chemistry, University of Copenhagen, Universitetsparken 5, 2100 Copenhagen, Denmark; 5 Laboratory for Biomolecular Network Dynamics, Biochemistry, Molecular and Structural Biology Section, Department of Chemistry, KU Leuven. Celestijnenlaan 200G box 2403, 3001 Heverlee, Belgium

## Abstract

Photoactivated localization microscopy (PALM) is a super-resolution imaging technique based on the detection and subsequent localization of single fluorescent molecules. PALM is therefore a powerful tool in resolving structures and putative interactions of biomolecules at the ultimate analytical detection limit. However, its limited imaging depth restricts PALM mostly to *in vitro* applications. Considering the additional need for anatomical context when imaging a multicellular organism, these limitations render the use of PALM in whole animals difficult. Here we integrated PALM with confocal microscopy for correlated imaging of the *C. elegans* nervous system, a technique we termed confocal correlated PALM (ccPALM). The neurons, lying below several tissue layers, could be visualized up to 10 μm deep inside the animal. By ccPALM, we visualized ionotropic glutamate receptor distributions in *C. elegans* with an accuracy of 20 nm, revealing super-resolution structure of receptor clusters that we mapped onto annotated neurons in the animal. Pivotal to our results was the TIRF-independent detection of single molecules, achieved by genetic regulation of labeled receptor expression and localization to effectively reduce the background fluorescence. By correlating PALM with confocal microscopy, this platform enables dissecting biological structures with single molecule resolution in the physiologically relevant context of whole animals.

Fluorescence microscopy techniques have greatly advanced our understanding of cell biology and physiological processes in living animals. However, diffraction of light limits the lateral resolution of conventional fluorescence microscopy to approximately 200 nm, whereas many subcellular components localize and interact at scales below this diffraction limit. To overcome this diffraction-imposed resolution barrier, several super-resolution imaging techniques have been developed over the past decade, including photo-activated localization microscopy (PALM)^1^,^2^, stochastic optical reconstruction microscopy (STORM)^3^, stimulated emission depletion microscopy (STED)^4^, structured illumination microscopy (SIM)^5^, super-resolution optical fluctuation imaging microscopy (SOFI)^6^, and derivatives thereof like dSTORM^7^, pcSOFI^8^, NASCA^9^, and S-PALM^10^. Together with the advantage of fluorescence microscopy where specific molecules can be detected with high contrast, super-resolution fluorescence microscopy has become a powerful tool to visualize biological molecules with subdiffractive resolution. Among these super-resolution techniques, PALM combines the advantages of single molecule detection with specific genetic labeling of bio-molecules, by expressing fusion proteins between the target of interest and a photoswitchable fluorescent protein (FP)^1^,^2^. Often used is mEOS2, a green-to-red switching FP, of which the switching rate can be controlled by UV-illumination^11^,^12^. Stochastic light-induced switching of these fluorescent proteins and detection of their single molecule fluorescence enables temporal separation of spatially overlapping molecules, for which precise coordinates can be calculated by fitting a 2D Gaussian function to the point spread function of the detected signal^13^. Depending on the signal to noise ratio and the subsequent precision of the fit, a typical resolution of 20 to 30 nm is achieved with PALM^1^,^2^.

Although commonly used for *in vitro* studies, super-resolution imaging techniques are maturing and now steadily being implemented to study more complex biological systems including whole animal models. Recent publications report on super-resolution fluorescence imaging in animals, through either Structured Illumination Microscopy (SIM) or Stimulated Emission Depletion (STED) microscopy^14^,^15^,^16^,^17^,^18^. However, due to limitations of the imaging depth, the application of single molecule based super-resolution microscopy, like PALM and STORM, remains challenging in intact animals. Single molecule detection requires the elimination of background fluorescence, for which PALM relies mostly on the total internal reflection fluorescence (TIRF) illumination mode. For objective-based TIRF systems, TIRF-mode illumination restricts the illuminated sample region typically above 200 nm from the coverslip surface, avoiding background fluorescence from regions at larger distances from the coverslip^19^. A recent study shows single molecule detection (SMD), just below the egg shell of *C. elegans* embryos using a quasi-TIRF approach, at depths of several hundreds of nanometres^20^. However, it remains to be proven that TIRF or sheet-based illumination can be adopted to obtain subdiffractive information from deeper lying tissues, like the nervous system, in animals. Overcoming this limitation would require a strict genetic control of labeled proteins, to limit the amount of fluorescent molecules present in out of focus regions and improve the depth penetration of PALM.

The small nematode model organism *Caenorhabditis elegans*, with its transparent body and ease of genetic manipulation, is highly amenable to whole-animal fluorescence microscopy, allowing observation of most of the animal with a high magnification objective. Its relatively simple nervous system counts 302 neurons and about 8,000 synaptic connections (Fig. 1a)^21^. With the complete nervous system anatomically mapped^21^, it provides a unique context to unravel neuronal functioning at the single molecule level. Neuronal signaling in *C. elegans* relies on a set of small molecule neurotransmitters similar to those found in other animals, including a major role for glutamatergic neurotransmission in mediating excitatory synaptic signaling^22^. Glutamate receptor subunit 1 (GLR-1) is one of at least two *C. elegans* genes homologous to vertebrate α-amino-3-hydroxy-5-methyl-4-isoxazole (AMPA)-type glutamate receptor subunits, which organize into tetrameric receptor complexes at postsynaptic sites^22^,^23^,^24^,^25^. GLR-1 is known to be implicated in the signal transduction of mechanosensory information and memory formation in *C. elegans*^24^,^25^,^26^,^27^,^28^,^29^,^30^. As the spatial pattern of the receptors correlates with their function in the neural circuit, it is crucial to link their single molecule distribution to specific neurons in the overall nervous system. Confocal laser scanning microscopy (CLSM) can deliver this kind of anatomical context, thus providing the framework for interpreting super-resolution data in a whole animal.

Here we introduce confocal correlated PALM (ccPALM), a method combining confocal with photo-activation localization microscopy, for single molecule mapping in *C. elegans*. This way, we visualized the distribution of GLR-1 clusters in the nematode’s nervous system with up to 20 nm resolution. To facilitate cell identification and to provide anatomical context for our PALM analyses^31^, we used transgenic worms expressing enhanced Green Fluorescent Protein (eGFP) in GLR-1–positive neurons. We then combined PALM with confocal microscopy to map glutamate receptor clusters onto specific neurons. By genetic regulation of the amount and localization of labeled GLR-1, our approach reduced the background fluorescence enabling TIRF-independent single molecule detection up to 10 μm deep. This regulation was achieved by choosing a promoter with limited expression throughout the body. Our ccPALM imaging study provides structural details on the molecular synaptic architecture, with the highest spatial resolution yet achieved with fluorescence microscopy in animals. Within the operation of neural networks, the number, size and dynamics of synapses are defining for the functional output. Combining our approach with knowledge on neural network functioning should help in interpreting single molecule based super-resolution data in *C. elegans*, and possibly other organisms. This will offer new opportunities for studying subdiffractive neural structures and plasticity.

## Results

### Photoconvertible labeling of GLR-1 proteins in *C. elegans*

**Figure 1:**
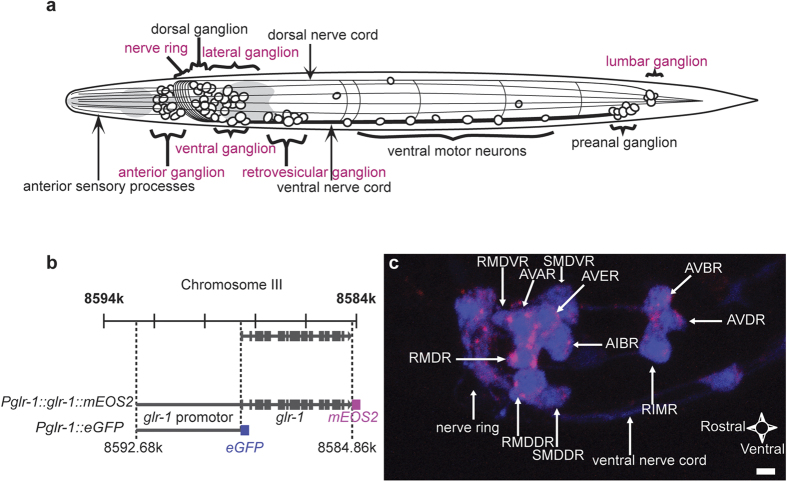
Labeling strategy for confocal correlated PALM. (**a**) Schematic overview of the *C. elegans* nervous system, showing the major neuronal ganglia and processes. Ganglia containing GLR-1 expressing neurons are marked in magenta. L1 larvae are approximately 250 μm long and 25 μm in diameter, but grow to 1.3 mm long and 80 μm diameter when reaching the adult stage. (**b**) Genomic position of the *glr-1*-gene on chromosome III. Boxes represent the exons, while the connecting bars represent the introns. Two amplicons were obtained by genomic PCR: the first containing the *glr-1* gene with approximately 4 kb of putative promoter sequence upstream from the start codon, and the second containing only the putative promoter region. The former was fused to the sequence encoding *mEOS2* while the latter was placed in front of the *eGFP*-encoding sequence. (**c**) Confocal image showing a partial Z-projection of the right lateral side of transgenic *C. elegans* expressing both the *Pglr-1::glr-1::mEOS2* and the *Pglr-1::eGFP* constructs. The GLR-1 expressing neurons are represented in cyan, while the localization of the GLR-1-mEOS2 fusion protein is coded in magenta. Annotated neurons indicated by white arrows. Scale bar measures 2 μm.

Our ccPALM strategy consists of genetically labeling and targeting proteins of interest, thereby limiting the expression of fluorescent fusion-proteins to certain regions in the animal. To visualize the distribution of GLR-1, we generated transgenic *C. elegans* that express GLR-1 labeled with the photoconvertible fluorescent protein mEOS2 (Fig. 1b). mEOS2 was fused to the cytoplasmic C-terminal tail of GLR-1, a region previously reported not to be essential for the functional activity of GLR-1 or GLR-2 tetramers when endogenous full-length GLR molecules are present as well^32^. Expression of this mEOS2-tagged GLR-1 was therefore introduced into a wild-type background and placed under the control of the *glr-1* promoter (Fig. 1b). We cloned the *glr-1* locus containing the promoter, intron and exon regions (Fig. 1b) into a modified pPD95.75 expression vector in which we substituted GFP by mEOS2. Although we could see red fluorescent signals by confocal microscopy in the head-region after illuminating the worm with UV-light, it was difficult to assign this fluorescence to certain neurons (Supplementary figure 1). To obtain this anatomical context information, we co-expressed transgenes for mEOS2-tagged GLR-1 and eGFP that were both under control of the *glr-1* promoter in *C. elegans* (Fig. 1b). Expression of eGFP was detected in several interneurons and motor neurons, consistent with previously reported patterns of *glr-1* expression^24^,^33^ (Fig. 1c & Supplementary movie 1). Following photoconversion of mEOS2 by UV-illumination, red fluorescent signals attributable to mEOS2-labeled GLR-1 appeared as punctate structures in the nerve ring, on the edge of the aforementioned neurons, and in the ventral nerve cord (VNC) (Fig. 1c). Localization patterns in the VNC were consistent with previously published physiological relevant GLR-1 distributions (Supplementary Figure 2)^32^.

### *C. elegans* immobilization for confocal correlated PALM

As PALM typically achieves a resolution of around 20 nm, any sample movement in that order of magnitude will render the resulting image useless. Therefore, we optimized sample-mounting protocols prior to PALM imaging. To this effect, we chemically fixed the worms with formaldehyde (or a combination of formaldehyde and glutaraldehyde) to prevent movement of the labeled receptors at the single molecule level. The worms, still in the fixation mixture, were placed on poly-L-lysine (PLL) coated imaging dishes. The fixation reagents likely also cross-linked surface proteins of the worms with the amino group of the PLL, thus anchoring the worms to the glass surface. After fixation, we embedded the worms in CyGEL, which immobilized them further and helped to minimize the refractive index mismatch between the media and the worm body; the refractive index of CyGEL is approximately 1.369 at 20 °C, which is slightly higher than the refractive index of water and closer to that of *C. elegans*^34^.

### Single molecule detection and analysis

**Figure 2:**
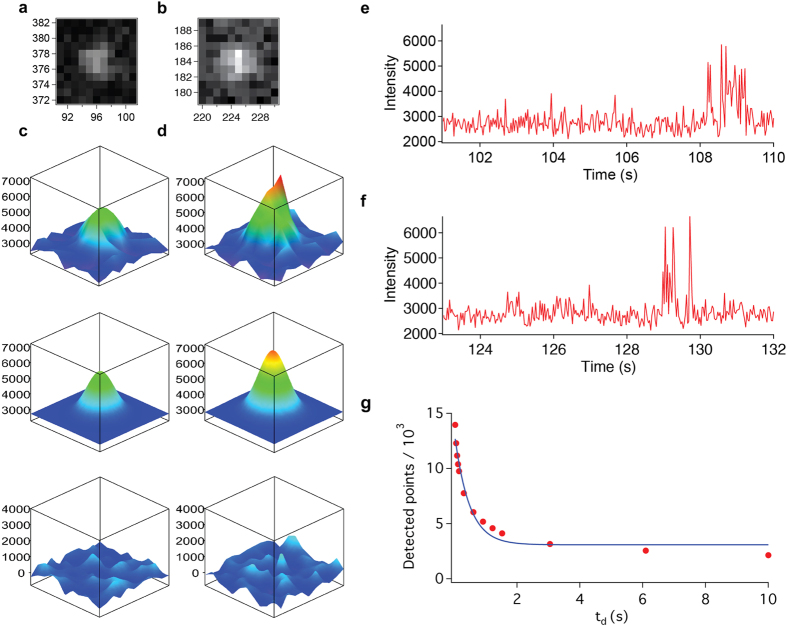
Single molecule detection in *C. elegans.* (**a,b**) Raw images (10 by 10 pixels) data showing 2 randomly chosen single molecules inside *C. elegans*. (**c,d**) 3D representation of the single molecule signal (top), 2D Gaussian fit (middle) and residuals after fitting (bottom) shown in (**a**) and (**b**) respectively. The y-axes on the 3D-plots denote the intensity in arbitrary units (**e,f**) Intensity time trace of the molecules shown in (**a,b**) indicating that mEOS2 blinks on and off multiple times, leading to possible multiple detections. (**g**) Graph showing the amount of detected unique molecules in function of the allowed dark time (t_d_) between detections (red circles). Exponential fit (blue line) shows the onset of a plateau at around 2 seconds.

To visualize the single molecule distribution of GLR-1, we subjected the mounted *C*. *elegans* to PALM imaging. Thanks to the restricted expression of mEOS2-labeled GLR-1 in only a small subset of neurons, background fluorescence was suppressed enough to detect respective single molecules allowing for PALM. We were able to detect single molecules under Köhler illumination up to approximately 10 μm deep (nominal focus position)^35^, although the background was reduced even further under highly inclined and laminated optical sheet (HILO) illumination^36^. We then sought to validate the quality of the recorded single molecule signals (Supplementary movie 2). The intensity-to-noise ratios of the detected molecules were mostly in the range of 6 to 8, allowing us to calculate their coordinates with an average accuracy of 20–30 nm^13^. After 2D Gaussian fitting using the Localizer software package^37^, over 24,000 spots could be detected in a representative PALM data set of a single worm (Fig. 2). Further analysis yielded the coordinates of these single molecules with an average accuracy of 25 nm (Fig. 2 panels a, b, c and d; Supplementary figure 3).

As mEOS2 molecules often fluoresce longer than a single camera frame acquisition^12^, they are usually visible in consecutive frames. We therefore regarded spots that appeared at the same location in consecutive frames as a single molecule^12^. However, a signal from one molecule might also temporally disappear due to the reported blinking behaviour of mEOS2 (Fig. 2 panels e and f), which results in the potential risk of counting a single molecule multiple times and generating artificial clusters in the final image^38^,^39^. When a molecule is detected in two non-subsequent frames on exactly the same position there are two possibilities: it is the same molecule that blinks, or it is a different molecule that was photoconverted at exactly the same place. When comparing randomly distributed mEOS2 molecules versus clustered mEOS2 molecules it was observed that these two possibilities occur at different time rates. When plotting the dark time (t_d_) vs the number of detected molecules, these 2 different rate constants can be seen as the biphasic function (Fig. 2g). The fast drop in the curve is the quick component which corresponds to the blinking of the mEOS2 molecules. The slow declining slope (after approximately 2.5 seconds) is the effect of mistakenly counting 2 different molecules as one). To circumvent the issue of overcounting, we allowed for a dark time (t_d_) of 10 seconds between discrete serial occurrences of a spot in order to attribute it as a specific molecule^39^.

In spite that GLR-1 is expected to cluster at postsynaptic regions, some non-clustered signals also appeared in the PALM image, inside as well as outside the neurons (Supplementary figure 4a). These signals probably arise from autofluorescent molecules, both inside and outside the neurons, and from non-targeted GLR-1 receptors or mEOS2 molecules cleaved from their target proteins, if located exclusively inside the neurons. To separate the GLR-1 clusters from this single molecule noise, we introduced a threshold based on the density of the molecules. Because synapses contain multiple molecules^40^, we filtered the image so only clusters would be shown and lone molecules removed. If a point had at least 4 neighbors in a 0.05 μm radius around the point, the point itself and its neighboring points would be retained (supplementary figure 4).

### PALM imaging of GLR-1 clusters in the ventral nerve cord

**Figure 3:**
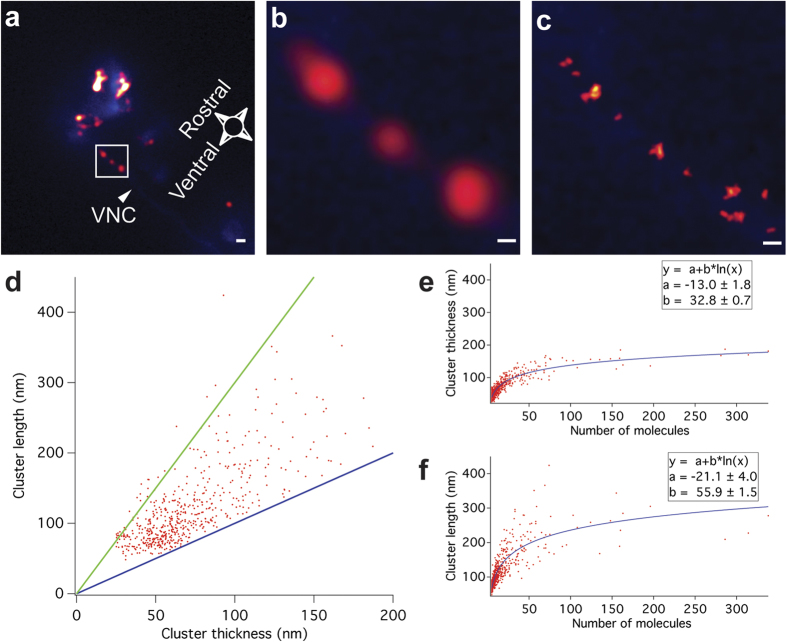
PALM of *C. elegans* VNC. (**a,b)** Head region of *C. elegans* with GLR-1 expressing neurons labeled with eGFP (blue) and GLR-1 molecules labeled with mEOS2 (red). eGFP fluorescence was recorded in standard epi-fluorescence mode and only clearly outlines the ventral nerve cord (VNC). Single GLR-1 molecules detected by PALM imaging were plotted as spots with a width of 214 nm to simulate diffraction-limited microscopy. When zooming in (**b**), GLR-1 puncta are visible in the VNC. (**c**) PALM image plotted at 25 ± 0.2 nm average resolution ± standard deviation, similar to (**b**), showing a more detailed structured of the GLR-1 puncta in the VNC. Scale bars measure 1 μm (**a**) or 250 nm (**b,c**). (**d**) Cluster thickness plotted versus cluster length (red dots). The function y = x is shown in blue, the function y = 3x is shown in green. Most clusters are situated between these lines. (**e**) Cluster thickness plotted as a function of number of molecules per cluster and (**f**) cluster length plotted as a function of number of molecules per cluster. Distributions shown (**e,f**) are fitted with a logarithmic function (blue curve). Function and fit coefficients ± s.d. are shown in box.

To demonstrate the capability of PALM for nanometre-precise protein localization in intact animals, we visualized the distribution of mEOS2-tagged GLR-1 in the VNC of *C. elegans* (Fig. 3). GLR-1 is reported to localize in a punctate pattern along these axon bundles^26^,^27^. We first located the focal plane containing the eGFP labeled VNC by wide-field fluorescence microscopy (Fig. 3 panels a,b and c, blue signals). Subsequent PALM imaging on the VNC yielded the distribution of GLR-1 with an average resolution of 25 nm (Fig. 3c). In addition, we reconstructed images that mimic the diffraction-limited images of GLR-1 puncta by rendering the PALM coordinates with a spot size of 214 nm (equivalent of 2 pixels) (Fig. 3 panels a and b). In these images, we observed puncta with a width of around 800 nm, consistent with the reported size by previous microscopy studies^27^,^30^. Although there are a larger number of puncta in the VNC, in this particular image only 3 puncta close to the head ganglia were in focus due to the shallow focal depth of the single molecule detection and the orientation of the worm.

In total we measured 117 individual worms, from which we discarded all datasets with too much sample drift, too high background, or a low number of detected molecules, leaving a remainder of 20 unique datasets to be analysed. These images revealed that a single punctum is often composed of multiple GLR-1 clusters with different sizes (Fig. 3c, supplementary figures 5 and 6). The VNC itself can be separated anatomically from both the co-injection marker in the intestine and the cell bodies in the head region, as they are sufficiently spaced apart when imaging the worm from the lateral side.

Since GLR-1 clusters in the VNC show varying sizes and shapes that are not always suitable for the use of common cluster analysis methods^41^,^42^, we implemented an analysis method previously used to analyse stretched synapses, (see materials and methods)^43^. Briefly, a 2D Gaussian was fitted over every cluster, providing us with the thickness, length and number of molecules per cluster. Over the 20 different datasets, 581 clusters present in the VNC were analysed. Of these we discarded 22 because they were smaller than our average resolution (25 nm) and thus not significant. When plotting the cluster thickness versus cluster length (Fig. 3d), we found that the majority of clusters range from round (length = thickness) to 3 times longer than thick. Furthermore, plotting the amount of receptors per cluster vs. cluster thickness yields a clear logarithmic distribution (Fig. 3e). Although cluster content increases, cluster thickness is limited to less than 200 nm. A similar plot of molecular content vs. cluster length (Fig. 3f) shows a more diffuse pattern, although still resembling a logarithmic distribution.

### Correlating PALM with confocal microscopy in whole animals

**Figure 4:**
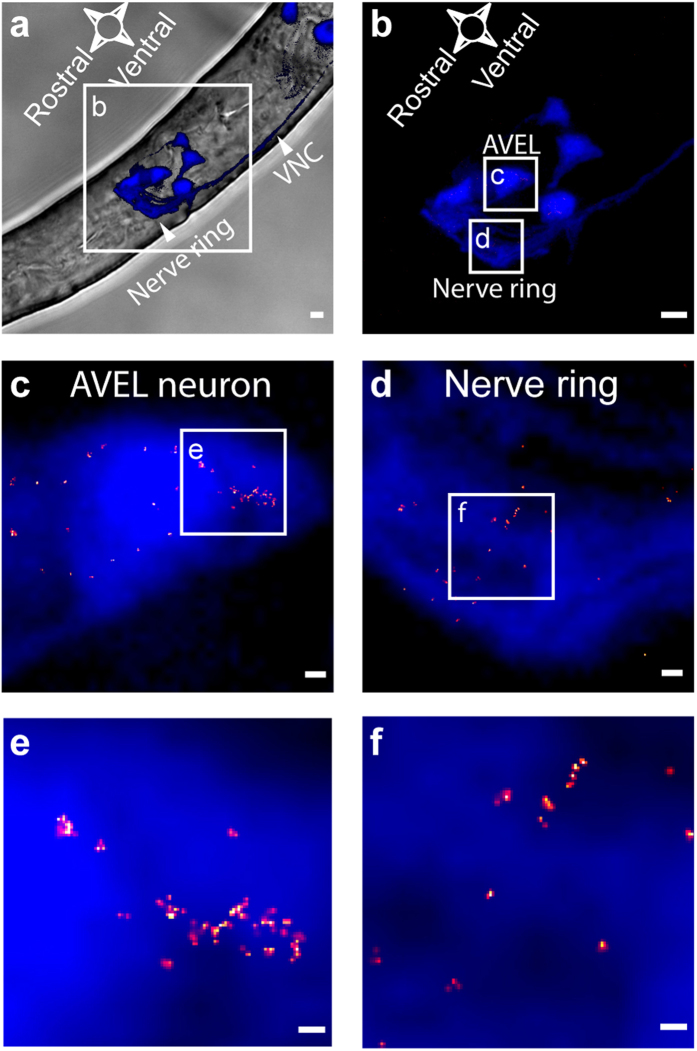
Confocal correlated PALM in *C. elegans*. (**a**) Partial confocal Z-projection (15 optical slices, total thickness of 5.30 μm) of eGFP fluorescence marking GLR-1 positive neurons and their processes (blue) overlaid with transmission image of the *C. elegans* head region (grey). White arrowheads indicate the nerve ring and the VNC. (**b**) Enlargement of GLR-1 expressing head neurons from Z-projection in (**a**). (**c,d**) Close up of the AVEL neuron (**c**) and the nerve ring (**d**) indicated in (**b**) with the distribution of GLR-1 mapped onto the neuron by ccPALM. (**e,f**) Close up of box in panels (**c**) and (**d**), respectively. Scale bars indicate 2 μm (**a,b**) 250 nm (**c,d**), or 100 nm (**e,f**). Cut-off resolution of PALM images (**c**–**f**) is 20 nm.

In addition to the GLR-1 distribution in axon bundles of the VNC, we aimed to determine the super-resolution pattern of GLR-1 in the densely packed neuronal head ganglia and the nerve ring (Fig. 1a). Due to the complex three-dimensional organization of neurons in these head regions, wide-field fluorescence microscopy did not suffice to distinguish individual eGFP labeled neurites and their cell bodies. As a consequence of the small depth-of-field typical for a high-magnification objective, out-of-focus fluorescence blurred the image (Fig. 3a). Confocal microscopy was specifically developed to circumvent this problem and is the standard imaging technique for thick fluorescently labeled biological specimens^44^. Therefore, we integrated PALM with confocal microscopy in an attempt to correlate the three-dimensional overview of GLR-1 expressing cells with the super-resolution pattern obtained by PALM. For this purpose, we first acquired a confocal Z-stack of the *C. elegans* head region, and annotated neurons that express GLR-1. The confocal image allowed us to adjust the focus to a focal plane-of-interest on which we subsequently performed PALM imaging. After analysis of the PALM dataset, we overlaid the single molecule distribution map of GLR-1 with the confocal image, thereby correlating the super-resolution data with its anatomical context (Fig. 4). For the left AVE interneuron (AVEL), we observed clusters of GLR-1 restricted to the outer edge of the cell soma that likely denotes the position of the plasma membrane (Fig. 4 panels c and e). We observed similar distributions for GLR-1 in other neurons, e.g. RIMR (Supplementary figure 7), and saw comparable clusters distributed throughout the nerve ring, where multiple synapses are expected to be present^21^ (Fig. 4 panels d and f).

## Discussion

Although super-resolution microscopy has become a routinely used technique in life sciences, its application to whole animals is still technically challenging and to the best of our knowledge, only a few reports are available. To date, STED microscopy and several modified SIM approaches have been successfully used to visualize neural or protein structures in living animals, with a resolution between 60 nm and 200 nm in three dimensions^14^,^15^,^16^,^17^,^18^. Hell’s group, for example, visualized the dynamic morphology and actin rearrangements of dendritic spines in living mouse brain using STED microscopy^14^,^17^. SIM-based approaches, using either multifocal or Bessel beam illumination, have been used to resolve cellular structures at embryonic stages of zebrafish, fruit fly, and *C. elegans*^15^,^16^. Compared to these methods, PALM requires considerably longer data acquisition times (typically a few tens of minutes)^2^ that impede dynamic imaging in living organisms. However, as it is based on single molecule detection (SMD), PALM achieves the highest image resolution among available super-resolution microscopy techniques. Previously, moving nanocrystals could be tracked inside *Drosophila* larvae and zebrafish embryos^45^. Another recent study on Single Particle Tracking reported SMD using near-TIRF illumination on the plasma-membrane just below the eggshell of *C. elegans* embryos (up to approximately 0.5 μm deep)^20^. In contrast, SMD at micrometre depths are required to probe neural structures and plasticity in most of the *C. elegans* larval or adult nervous system. The single molecule imaging approach reported here combines TIRF-independent PALM with confocal microscopy, and is capable of visualizing protein distribution – with nanometre resolution – within the multicellular context of an intact animal up to 10 μm deep.

A key feature of our method is the TIRF-independent SMD, based on limiting the background fluorescence by genetically controlling the mEOS2-tagged protein pool. Labeled proteins of interest should ideally be expressed at low levels in a limited number of cells. GLR-1, the protein used in this study, matched these requirements and we were able to perform SMD deep in the animal by using HILO or wide-field illumination. We expect that our method can also be applied to proteins present (in low amounts) in an abundant number of cells; placing their genes under control of a promoter that restricts expression to a limited number of cells of interest, could provide sufficiently low background levels in this case. Our ccPALM strategy is however not yet appropriate for proteins that are expressed abundantly throughout a cell, as this might result in too low signal-to-noise ratios.

Confocal laser scanning microscopy (CLSM) has long been the golden standard for biological imaging, as it removes out-of-focus fluorescence with a pinhole, thus enabling optical sectioning of thick biological specimens^44^. Although labeling of neuronal structures with eGFP could allow distinguishing rough anatomical features by wide-field illumination, such as the *C. elegans* VNC, identifying single neurons and their processes requires CLSM. Integrating this technique with TIRF-independent PALM in model organisms gives us the opportunity to study protein distributions in annotated parts of the nervous system, and potentially other cell types. In addition, here we only used single colour ccPALM to highlight all GLR-1 expressing cells, but the method can be expanded to multicolor correlated imaging, for example, to co-visualize pre- and postsynaptic neurons. This should allow identification of specific synapses between two distinct neurons and study their plasticity.

The neural circuits of the *C. elegans* nervous system are modulated by environmental cues, resulting in behavioural plasticity including learning behaviour. The worm’s escape response shows long-term habituation to repeated mechanical stimulation^24^,^25^, which correlates with decreased sizes of an invariant number of GLR-1 puncta in the VNC^28^. We revealed with PALM that these puncta are composed of multiple clusters of GLR-1, and super-resolution imaging could further prove invaluable in unravelling the exact changes in size and/or number of synapses in these puncta underlying learning behavior.

In addition GLR-1 is thought to form functional heteromultimers with GLR-2. In *C. elegans*, GLR-2 delivery to synapses is mediated by kinesin (UNC-116/KIF-5), and kinesin-defective worms showed brighter puncta and diminished gating^46^. Super-resolution experiments in *C. elegans* with GLR-1 and GLR-2 could be interesting in dissecting the mechanisms of multimer formation on a molecular level. Our results show that clusters are elongated, as previously observed in cultured rat hippocampal neurons^43^. Their length ranges from 1 to 3 times longer than their thickness. We also see that cluster thickness is a logarithmic function of its molecular content, with a maximum of around 200 nm. This distribution can thus only be examined by super-resolution microscopy. Cluster length shows a less clear relation with its content, however the maximum cluster length is below 450 nm. Perinuclear clustering of GLR-1 probably reflects an intermediate in GLR-1 trafficking from its site of synthesis in the cell body to axonal regions, which is thought to play a key role in synaptic maintenance and plasticity^46^,^47^,^48^. As electron microscopy can only estimate the PSD-size, one needs to rely on super-resolution microscopy techniques like PALM for assessing the size of receptor clusters. Previous super-resolution experiments on *in vitro* rat brain cells showed clusters of GluRAs, the mammalian orthologs of the *glr* genes. GluRA clusters the size of around 70 nm^43^,^49^, and heterogeneous distributions of GluRA across a larger area in the synapse up to approximately 300 nm^50^ have been reported. The neurons of nematodes are morphologically different from mammalian neurons; *C. elegans* do not have typical synapse-containing neuronal spines, but form synapses en passant on its axons. Therefore, AMPAR clusters of *C. elegans* are not expected to exactly match the mammalian cluster morphology, even though the cluster size and the area of the GluRA distribution reported there are comparable to our results in the *C. elegans* ventral nerve cord. The clusters of around 50 nm in diameter are similar to the size of isolated rat synaptic transport vesicles measured by electron microscopy^51^, although cluster size varies greatly and the cluster shape ranges from round to stretched. As the synapses are distributed in a three-dimensionally organized neural system, we might be observing a synapse from the side, explaining the stretched shape^50^. Although the number of molecules per cluster varies greatly, the average number of molecules per cluster is 20.2 (±33.4 s.d.) with 92.7% of all clusters containing less than 50 molecules. Maximal observed content was 338 molecules in a single cluster, however we can not rule that 2 clusters arranged along the z-axis show up as 1 larger cluster in the image, something which could be assessed in the future by 3D-PALM. The average of 20 molecules per cluster is comparable to previous results using antibody based labeling in rat hippocampal neurons, although neither method labels all present receptors with complete certainty and probably slightly underestimates the exact number of receptors^43^. In our case also endogenous unlabeled receptors are present, while in the other case antibodies might not bind to every receptor.

In the case of L1 larvae, we could detect single molecule signals up to 10 μm deep (nominal focus position) in the organism, which is about half of the diameter of the worm. SMD could be achieved at greater depths in larger larvae pointing to a refractive index mismatch at the surface of the worm that might produce lens effects. Although we embedded the worms in CyGEL to match their refractive indexes, a remaining mismatch due to heterogeneity of this parameter inside the worm likely perturbs the detection of single molecules. Further optimization of the media and the implementation of adaptive optics^52^,^53^,^54^ could compensate the sample-induced aberrations to enable deeper imaging by PALM and thus cover the full diameter of *C. elegans*. Other interesting challenges lie in the implementation of 3D-PALM^55^,^56^,^57^,^58^, whether or not in combination with a light-sheet based illumination approach^59^. This way we can estimate the clusters’ elongated shapes and its distributions in 3D. We anticipate that ccPALM will complement the growing number of biological super-resolution microscopy techniques and help to elucidate physiological and neurobiological relevant structures and interactions in super-resolution, inside intact animals.

## Materials and Methods

### Plasmid construction of *Pglr-1::glr-1::mEOS-2*

The gene encoding the mEOS2 fluorescent protein^11^ (www.addgene.org plasmid nr 20341), was amplified by PCR with a forward primer containing an *AgeI* site and a revers primer containing a *EcoRI* site (primer sequences in Supplementary Table 1) using Pfu-polymerase (Fermentas). The commonly used pPD95.75 reporter vector (kindly provided by A. Fire, Stanford University, Stanford, USA) was modified by swapping the GFP S65C-coding sequence by the *mEOS2*-gene at the *AgeI*/*EcoRI* site. Correct insertion of the gene, yielding the pPD95.75_mEOS2 plasmid, was confirmed by sequence analysis (LGC Genomics). The *glr-1* locus including all exons and introns and approximately 4kb of putative promoter sequence upstream from the start codon, was amplified using genomic PCR (High Fidelity PCR enzyme mix; Fermentas) by which restriction sites and a linker were added (primers see Supplementary table 1). Amplicons were cloned into the *XbaI/SmaI* site of the pPD95.75_mEOS2 vector, yielding a *Pglr-1::glr-1::mEOS-2* transgene. Sequence analysis confirmed the correct insertion of the glr-1 gene and the sequence of the linker ensuring in-frame translation to yield a full-length GLR-1_mEOS2 fusion protein

### Plasmid construction of *Pglr-1::eGFP*

GFP S65C in pPD95.75 was swapped by eGFP at *AgeI/EcoRI*, yielding a pPD95.75_eGFP variant. 4 kb upstream of the start codon of *glr-1* was amplified by genomic PCR to obtain the promotor region (*Pglr-1),* which was cloned into the *XbaI/SmaI* site of the pPD_95.75_eGFP, yielding the *Pglr-1::eGFP* transgene.

### *C. elegans* strains and culture conditions

*C. elegans* were cultured at 15 or 20 °C under standard conditions and fed *E. coli* OP50^60^. Wild type animals were Bristol variety N2 (obtained from the *Caenorhabditis* Genetics Center). The following transgenic strains were used: LSC79, *lstEx181-lstEx186 [Pglr-1::glr-1::mEOS2; Pelt-2::gfp].* LSC411-LSC412, *lstEx335-lstEx344 [Pglr-1::glr-1::mEOS2; Pglr-1::egfp; Pelt-2::gfp]*.

### Formation of transgenic *C. elegans* strains

Germline transformations were carried out by standard microinjection techniques as described previously^61^. The plasmids containing *Pglr-1::egfp* and/or *Pglr-1::glr-1::mEOS2* were injected into wild type animals at 20 or 30 ng/μl, respectively, using the *Pelt-2::gfp* gene as coinjection marker and L4440 as carrier DNA. Final concentration of total injected DNA was 110 ng/μl.

### Confocal microscopy and cell identification

Cellular expression of the *Pglr-1::gfp* transgene was visualized with an inverted laser scanning microscope (Fluoview FV1000; Olympus, Tokyo, Japan). *C. elegans* were harvested by washing worms off a culture plate with M9-buffer. Worms were washed (2-3 times) with M9-buffer to remove excess of food^62^. Samples were prepared by sedating the worms with NaN_3_ and immobilizing them by sandwiching them between a coverslip and a 1 to 2% agarose patch. Cells in L1 larvae were visualized by Normarski differential interference contrast (DIC) microscopy and identified based on their position and morphology. Z- projections of confocal images were produced by Olympus Fluoview Viewer v3.0.

### Fixation and immobilization of *C. elegans* for PALM

For PALM, the worms were harvested similarly as in the case of confocal microscopy. They were then fixed in a 4% formaldehyde (Thermo Scientific) or 4% formaldehyde+0.2% gluteraldehyde (Sigma-Aldrich) solution in phosphate-buffered saline (PBS) buffer, the latter resulting in a neglectable fraction of mobile single molecules during PALM imaging^63^. The mixture of fixation agents and worms was then incubated on densely Poly-L-lysine (Sigma-Aldrich) coated imaging chambers (P35G-0-10-C; MatTek Corporation) to promote the attachment of worms on the coverslip, probably through the crosslinking of the worm with Poly-L-Lysine. The fixation mixture was removed and the worms were quickly immersed in 50–150 μl CyGel^TM^ (BioCompare) to immobilise them, match the refractive index and prevent the samples from drying out. The outer ring of the imaging dish was filled with approximately 1 ml 1-2% agarose as to further prevent drying of the sample by saturating the vapour pressure inside the chamber.

### PALM microscopy set-up

The homebuilt microscope consists of an Olympus IX71 or IX83 inverted microscope body, equipped with an oil-immersion objective lens (PlanApo TIRF 60X oil, NA = 1.45 Olympus). Illumination was provided by a Coherent Sapphire 561 nm, 100 mW DPSS laser, a Coherent Sapphire 488 nm, 100 mW DPSS and a Coherent Cube 405 nm, 100 mW diode laser (attenuated with filters of 2.5 to 3 Neutral Density). The different laser lines were combined using a zt561 bcm, a zt488 bcm and a zt405 bcm (Chroma Technology Inc.) The laser light was treated to be circularly polarized and the beam was expanded 10 times before being guided to the sample by a z405/488/561rdc dichroic (Chroma Technology Inc.) and focused on the back aperture of the objective, to ensure collimated and uniform illumination of the sample. Detection was done by projecting the fluorescence image leaving the side port of the microscope with a 2.5X projection lens (Olympus) onto the CCD-chip of a Hamamatsu C9100–113 EM-CCD camera, after being filtered with a 572 long pass (HQ572) and a 590/40 band pass (HQ590/40-2P) emission filters (Chroma Technoloy Inc.). 405 nm laser power was gradually increased during the measurement to ensure a stable number of mEOS2 conversions per unit of time, until almost no photoconversion was observed anymore, as to avoid undercounting due to incomplete photoconversion (Supplementary figure 8). The final pixel size of the acquired image was 107 nm. Analysis was done using the Localizer-software^37^, using the following parameters: “GLRT segmentation algorithm”, “8-way adjacency particle finding”, “PSF standard deviation”: 1.8 pixels and “GLRT insensitivity”-values between 25 and 30. Wide field and PALM images were overlaid using the same software.

The Localizer software has an implemented drift correction algorithm^64^, which does not use fiduciary markers. Briefly, the complete set of localized positions was divided into non-overlapping subsets containing X positions, where the first subset contained all emitters localized at the beginning of the movie, the last subset contained all emitters localized at the end of the movie. All other emitters were distributed over these batches according to the frame in which they were detected. For each of the batches, a localization image was calculated by creating a 2D histogram, where each emitter was assigned to a bin based on its x and y coordinate. The bin of the histogram was calculated and the relative shift between subsequent localization images was then estimated with subpixel precision by calculating the 2D phase correlation and fitting the resulting maximum to a 2D Gaussian function. The consecutive drift estimations were then interpolated using a polynomial fit and used to determine the approximate position of the sample relative to the starting point at each time point. The resulting offset was then subtracted from the fluorophore positions to obtain the drift-corrected positions. The drift correction makes use of all molecules that have been detected by the fitting procedure. If a molecule varies in intensity in subsequent frames and is not bright enough (low SNR) in some of these frames, it will not be fitted in those frames and thus its position in those frames will not be used for the drift correction.

Single molecule filtering was performed using the localizer “remove outlier position” function. Used parameters where “4 neighbors” in a “0.5 pixel radius”, using the option to retain all molecules in ‘positive’ radii, as not to remove the edges of smaller clusters. This operation can be thought of as similar to changing the contrast in a confocal image: low intensity background in a confocal image is analogous to sparsely distributed molecules in PALM images, as molecular density of fluorophores is linked directly to fluorescence intensity.

### Cluster analysis

Most observed clusters appear oval shaped and are thus not very good candidates for commonly used cluster analyses like Ripley’s K function or the ‘pairwise correlation’ algorithm.

We fitted all clusters (containing at least 5 molecules) with a 2 dimensional Gaussian function, as done before^43^,^65^. This function was allowed to rotate freely to accommodate any orientation the cluster might have. The x and y standard deviations were not coupled, allowing it to fit elongated structures. Every visible cluster was contoured by the operator and the fit was applied. Every cluster yielded following parameters: Number of molecules present, coordinate of cluster center, X-standard deviation of Gaussian, Y-standard deviation of Gaussian. This novel routine is now part of the open-source software Localizer. To translate the standard deviation to a length scale, we took the full width half maximum (FWHM) of the Gaussian, corrected for the actual pixel size, as the edge of the cluster. All data were organized so the X-length was smaller than the Y-length, yielding respectively the cluster thickness and the cluster length. All clusters with an observed cluster thickness of less than the resolution (25 nm) were removed.

### Correlated Confocal PALM measurements

Combined confocal microscopy and PALM were done on a Carl Zeiss Elyra PS.1 equipped with an LSM780 module (kindly provided by Carl Zeiss Microscopy, Jena, Germany). The system was equipped with an alpha Plan-apochromat 100X NA = 1.46 oil objective. The combination of a 561 nm laser with a NA = 1.46 objective, gives us a theoretical depth-of-field of 832 nm. Final pixel size was 100 nm. Analysis was done using the Carl Zeiss Zen 2012 software (black edition v8.0), with following parameters for PALM: a ‘peak mask size’ of 9 pixels, ‘peak intensity to noise’ of 7, the fit model ‘x,y 2D Gaussian fit’, and ‘average before localization’ with an ‘off gap’ of 200 frames in a ‘capture radius’ of 1 pixel. Resulting PALM and confocal images were overlaid using FIJI (www.fiji.sc). Resulting image was further overlaid with transmission image using Adobe Photoshop CS6, using the ‘select - color range’ function, with ‘fuziness’ filter to remove the black confocal background. Confocal/transmission and PALM images differ 0.72 nm in pixel size, however the maximum resulting error at the image edges when overlaying is less then the diffraction limit, thus we did not correct for this negligible difference in zoom-factor.

## Additional Information

**How to cite this article**: Vangindertael, J. *et al.* Super-resolution mapping of glutamate receptors in *C. elegans* by confocal correlated PALM. *Sci. Rep.* **5**, 13532; doi: 10.1038/srep13532 (2015).

## Supplementary Material

Supplementary Movie 1

Supplementary Movie 2

Supplementary Information
