# Corrigendum: Super-resolution mapping of glutamate receptors in *C. elegans* by confocal correlated PALM

**DOI:** 10.1038/srep26047

**Published:** 2016-05-20

**Authors:** Jeroen Vangindertael, Isabel Beets, Susana Rocha, Peter Dedecker, Liliane Schoofs, Karen Vanhoorelbeke, Johan Hofkens, Hideaki Mizuno

Scientific Reports 5: Article number: 13532; 10.1038/srep13532 published online: 09012015; updated: 05202016.

The original version of this Article contained a typographical error in the spelling of the author Karen Vanhoorelbeke, which was incorrectly given as Karen Vanhoorelbeeke. This has now been corrected in the PDF and HTML versions of the Article.

