# Supplementary material for: Super-resolution mapping of glutamate receptors in C. elegans by confocal correlated PALM
Source: Sci Rep. 2015 Sep 1;5:13532. doi: 10.1038/srep13532 (PMC4555104; doi:10.1038/srep13532)
Supplement: Supplementary Information [file srep13532-s3.pdf]

Supplementary information for the manuscript entitled:

Super-resolution mapping of glutamate receptors in *C. elegans* by confocal correlated PALM

Authors:

Jeroen Vangindertael[1,3,5], Isabel Beets[2], Susana Rocha[1,5], Peter Dedecker[1], Liliane Schoofs[2], Karen Vanhoorelbeeke[3], Johan Hofkens[1,4] and Hideaki Mizuno[5]*

*: corresponding author. Email: Hideaki.Mizuno@chem.kuleuven.be

Affiliations:

1) Laboratory for Photochemistry and Spectroscopy, Division of Molecular Imaging and Photonics, Department of Chemistry, KU Leuven. Celestijnenlaan 200F, 3001 Heverlee, Belgium
2) Laboratory for Functional Genomics and Proteomics, Division of Animal Physiology and Neurobiology, Department of Biology, KU Leuven. Naamsestraat 59, 3000 Leuven, Belgium.
3) Laboratory for Thrombosis Research, Interdisciplinary Research Facility Life Sciences, KU Leuven Kulak. E. Sabbelaan 53, 8500 Kortrijk, Belgium
4) Nano-Science Center, Department of Chemistry, University of Copenhagen, Universitetsparken 5, 2100 Copenhagen, Denmark
5) Laboratory for Biomolecular Network Dynamics, Biochemistry, Molecular and Structural Biology Section, Department of Chemistry, KU Leuven. Celestijnenlaan 200G box 2403, 3001 Heverlee, Belgium.

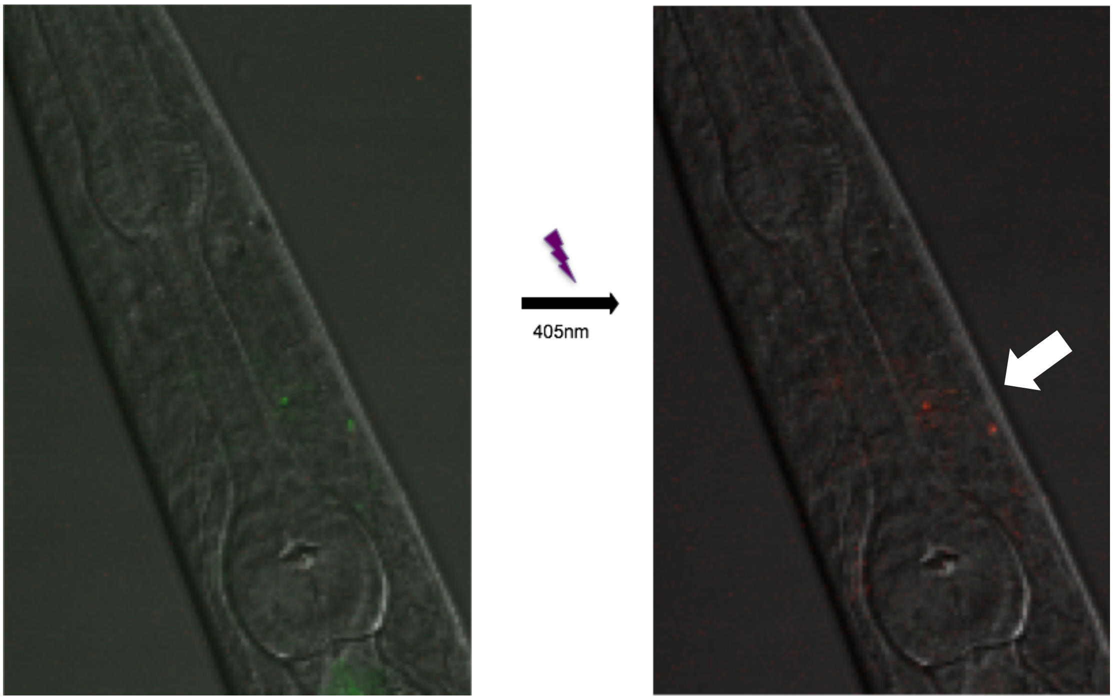

Supplementary Figure 1
Photoconversion of mEOS2 in *C. elegans.*

C. elegans only expressing the *Pglr-1::glr-1::mEOS2* transgene. Both images are a single plane obtained by CLSM. On the left is the worm before UV-illumination, on the right is the same worm after UV illumination. The mEOS2 FP fused to GLR-1 (present between the 2 pharyngeal bulbi) is photoconverted from the green to the red form (white arrow). Furthermore it can be seen that it is difficult to assign the mEOS2 signals to specific neurons. Images are overlaid with DIC-image of the worm (grey).

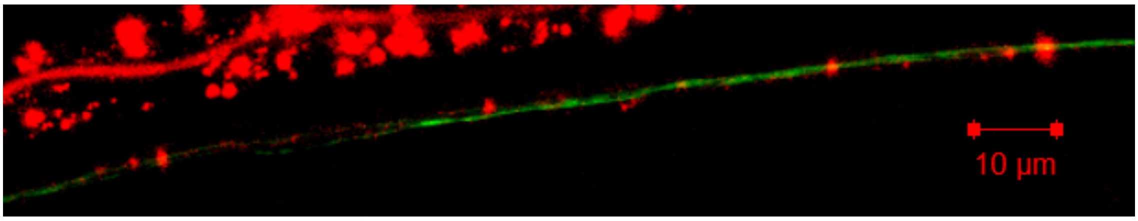

Supplementary Figure 2
GLR-1 expression pattern in VNC

Confocal image of the ventral side of a C. elegans worm expressing Pglr-1::glr-1::mEOS2 and Pglr1::eGFP transgenes. VNC is shown in green, GLR-1 present in the VNC is shown in red. Red signals outside the VNC are gut-associated autofluorescence GLR-1 clusters are organized in a repetitive manner, very similar to the previous reported function GLR-1[1]. This shows that the observed clusters are physiologically relevant.

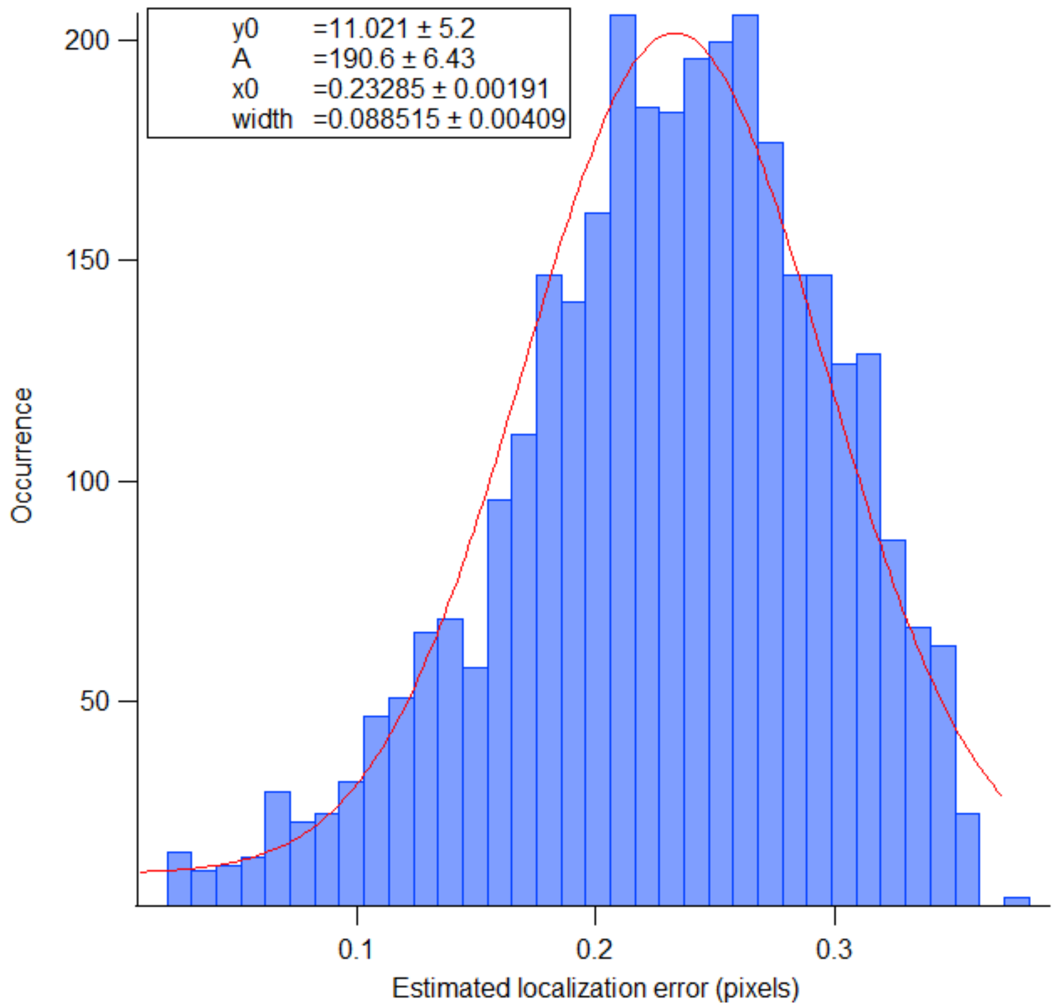

Supplementary figure 3
PALM Localization precision of Fig 3

Estimated localization error plotted versus number of detected molecules. Pixel size was 107 nm, yielding an average estimated resolution of 24,9 nm ± 0.2 (s.d.). 2D-gaussian fitting was done by the Localizer-software.

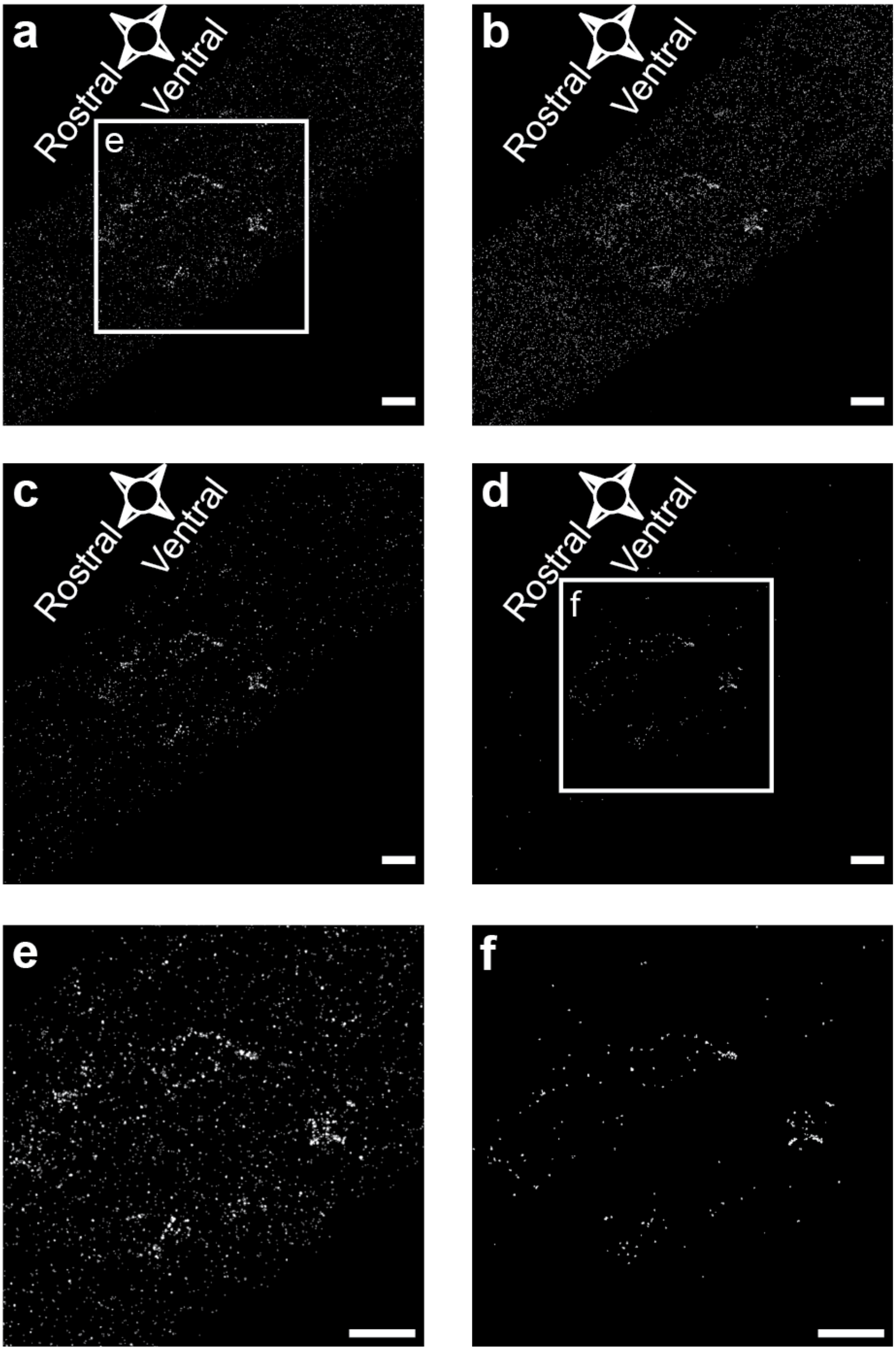
a
Rostral
Ventral
e
b
Rostral
Ventral
c
Rostral
Ventral
d
Rostral
Ventral
f
e
f

Supplementary figure 4
PALM image processing & filtering algorithms.

(a) PALM data from Figure 4, plotted without any filtering. Although some structures can be distinguished within the noise, they are not very sharp. **(b)** Consolidation of blinking molecules. mEOS2 molecules can 'blink' many times. This means they switch reversibly between a non-fluorescent and a fluorescent state. This photophysical behaviour can have the consequence that one unique molecule is detected multiple times during the PALM experiment. When this happens, artificial clusters can appear in the analyzed result. To prevent the overcounting of a single molecule we allow molecules to have an 'off-time' of 10 s between 2 fluorescent events (see Figure 2). This operation greatly reduces the chance of counting the same molecule multiple times. Although it appears that the noise in panel (b) is enhanced, this in is reality not the case; the overcounting in panel (a) leads to a larger dynamic intensity range, appearing as an artificial contrast enhancement. **(c)** Single molecule noise reduction. By removing single molecules that are not part of any structure (at least 4 detected single molecules need to be present in a 50 nm radius in order to be plotted) we can remove a large part of the noise. **(d)** By both the consolidation (b) and filtering (c) algorithms, we can remove both single molecule noise and clustering artifacts, leaving only the real signals. **(e)** and **(f)** Expanded view of panels (a) and (d). Here the effect of the combined algorithms can be seen clearly.
All analyses were done with Carl Zeiss Zen-software v8.0. Only molecules with a localization accuracy of 20 nm or better, are shown. Scale bars represent 2 µm.

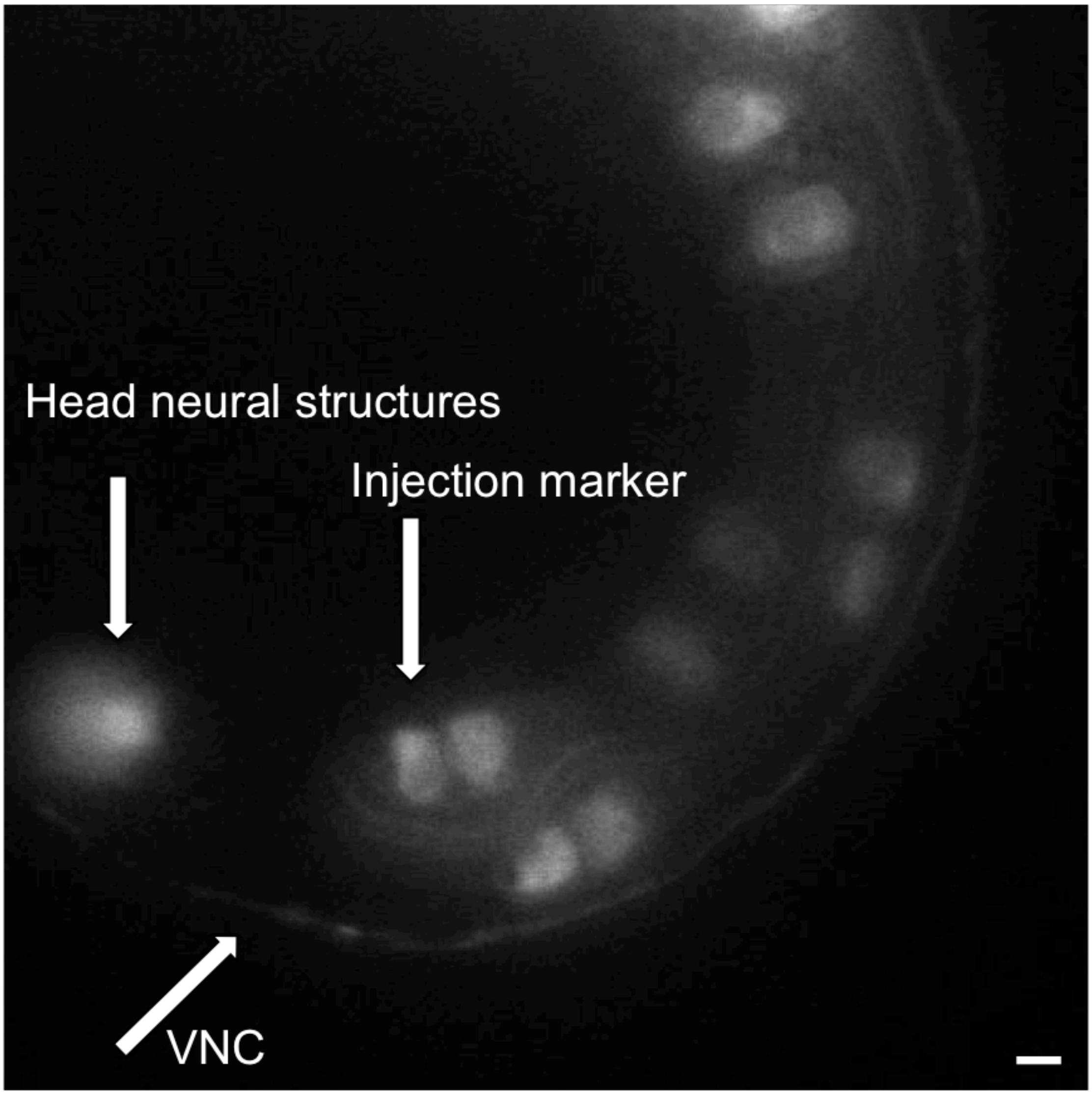

Supplementary figure 5A
Wide-field image of *C. elegans* expressing eGFP in the VNC. The VNC (bottom) is in focus over the whole field of view. On the left hand side some blurry out of focus neural structure can be seen. The fluorescent cells throughout the rest of the body are co-injection marker expressing gut cells. Scale bar is 2 µm.

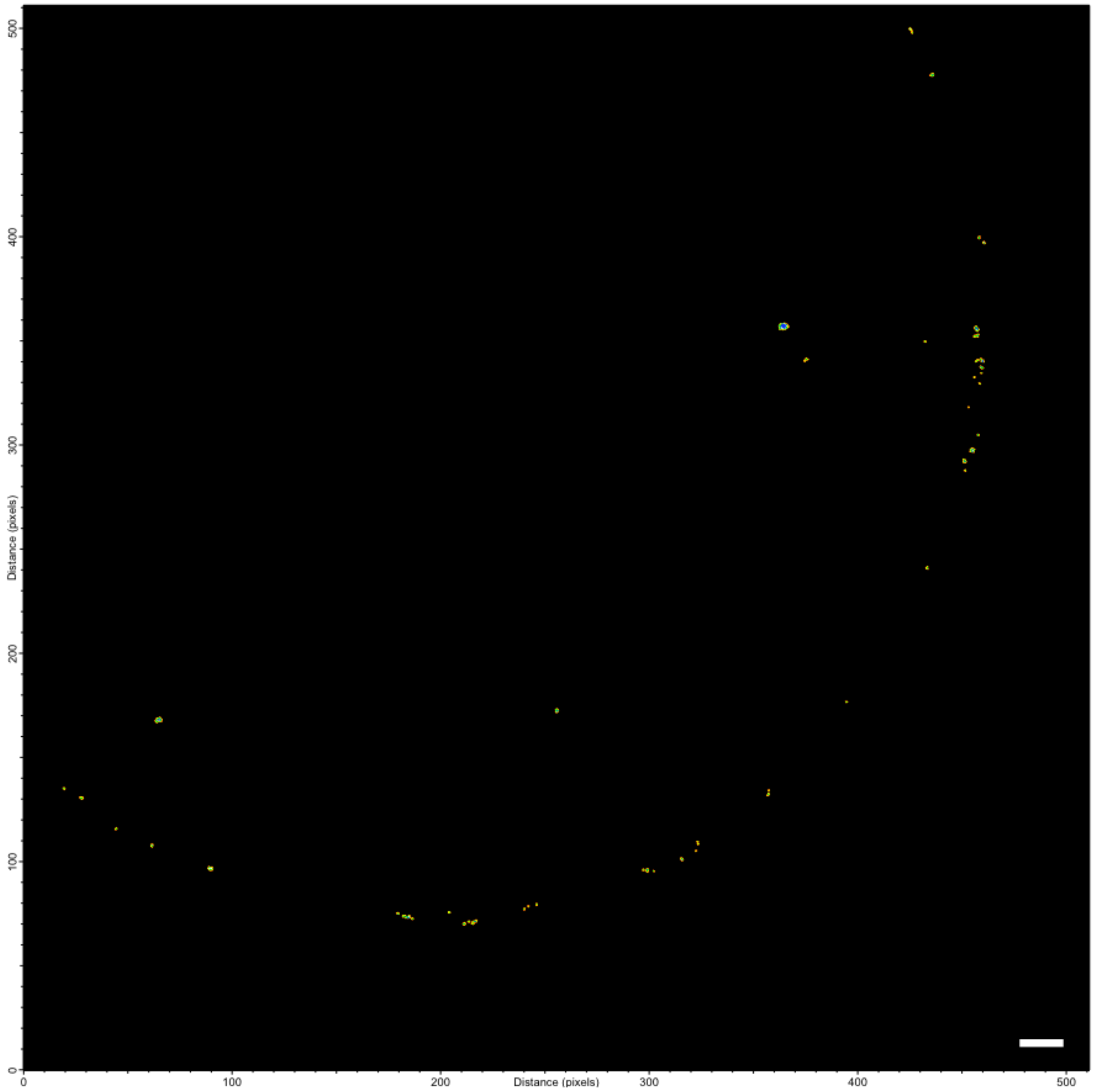

Supplementary figure 5B

Superresolution image showing GLR-1 organized in clusters inside the VNC (supplementary figure 5A). Autofluorescence inside the rest of the body is almost completely removed by the applied filtering techniques. Average PALM resolution is approximately 25 nm. Scale bar is 2 µm.

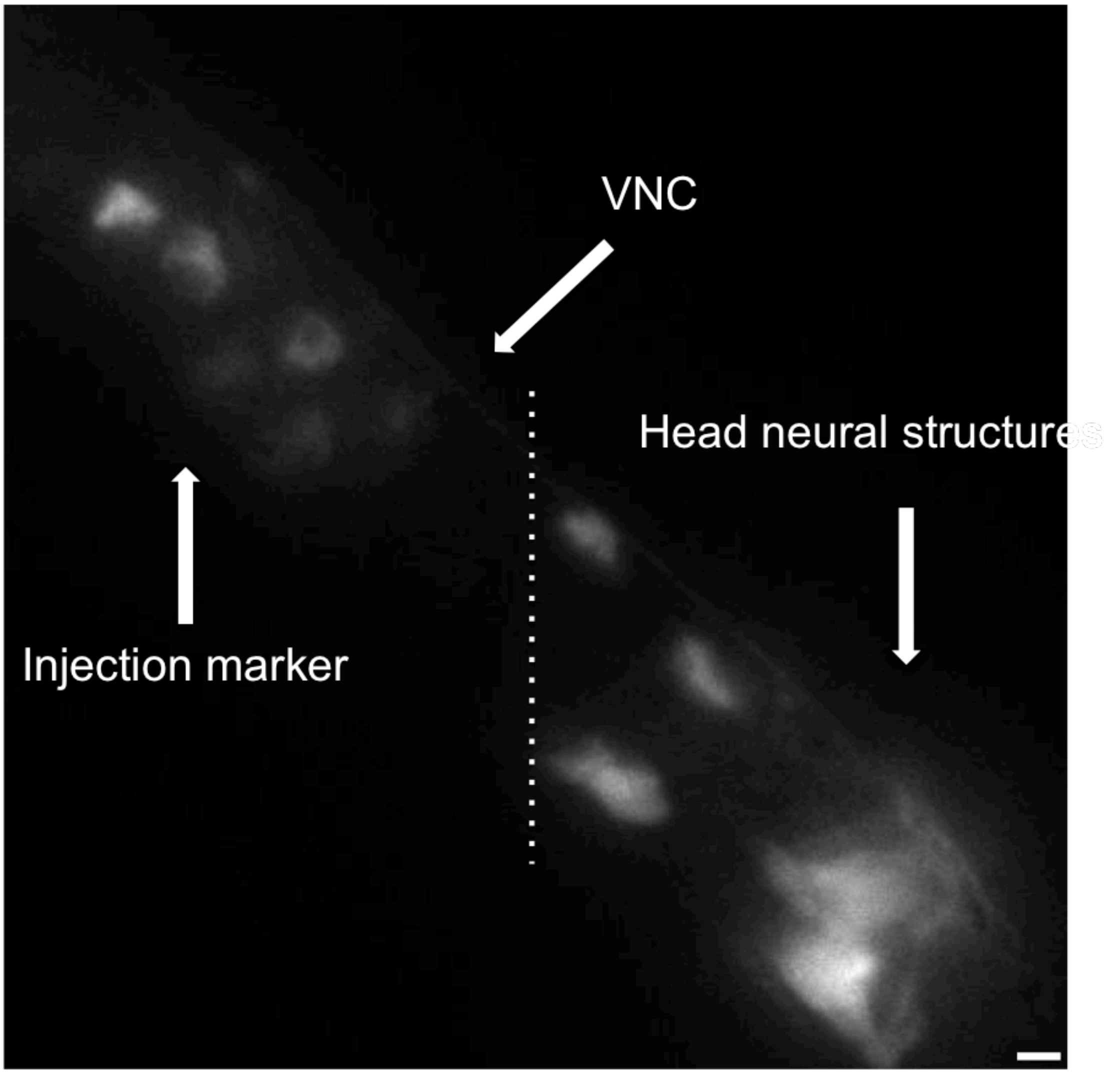

Supplementary figure 6A
Wide-field image of *C. elegans* expressing eGFP in the VNC and multiple neurons. The VNC (top) is in focus over the whole field of view. On the right hand side multiple neurons and some blurry neural structures, probably the nerve ring combined with some out of focus cells, can be seen. The fluorescent cells throughout the rest of the body (left hand side) are co-injection marker expressing gut cells. Scale bar is 2 µm.

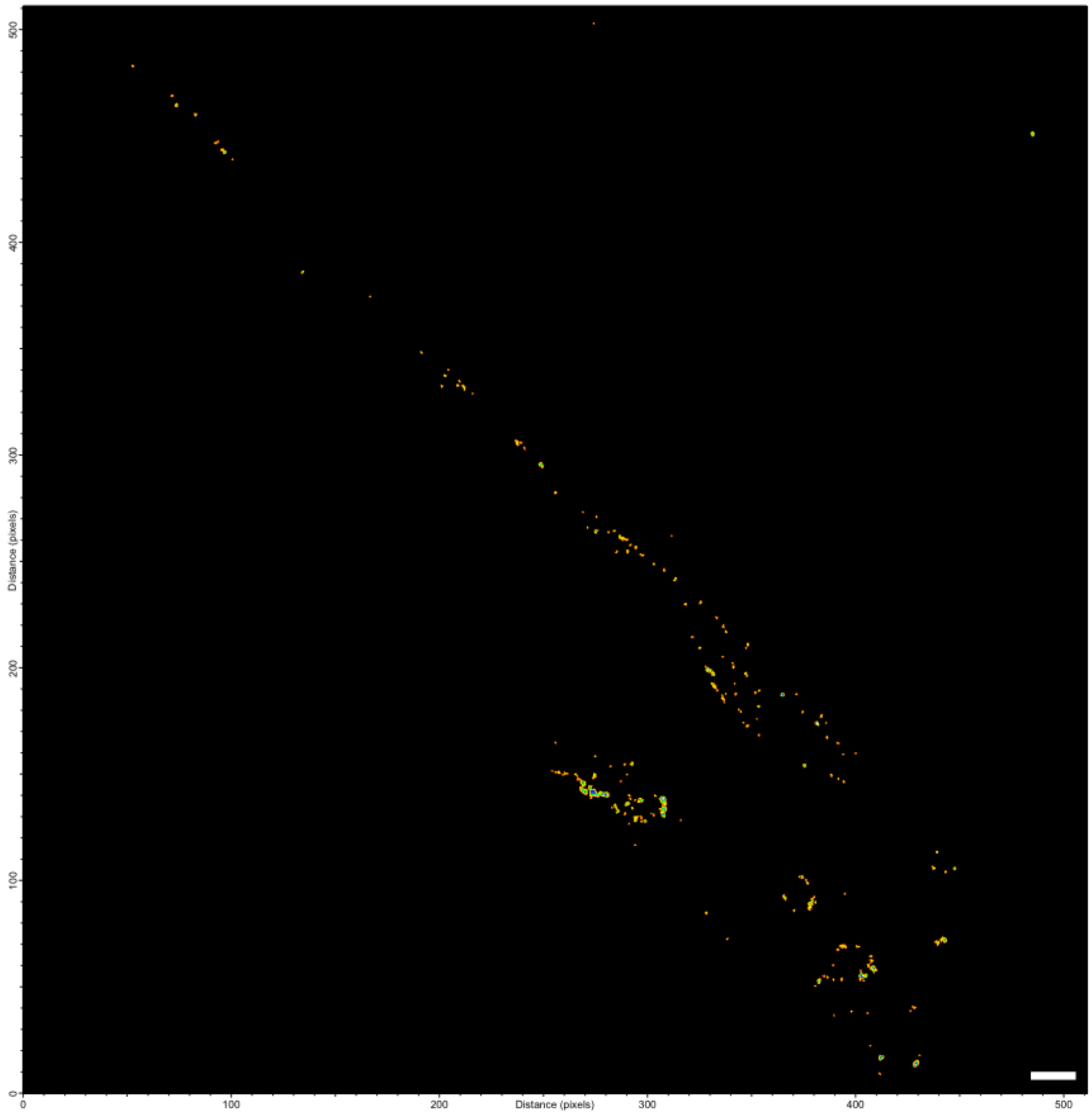

Supplementary figure 6B

Superresolution image showing GLR-1 organized in clusters inside the VNC (supplementary figure 6A). Also GLR-1 inside neuronal cell bodies can be seen. Autofluorescence inside the rest of the body is almost completely removed by the applied filtering techniques. Average PALM resolution is approximately 25 nm. Scale bar is 2 µm .

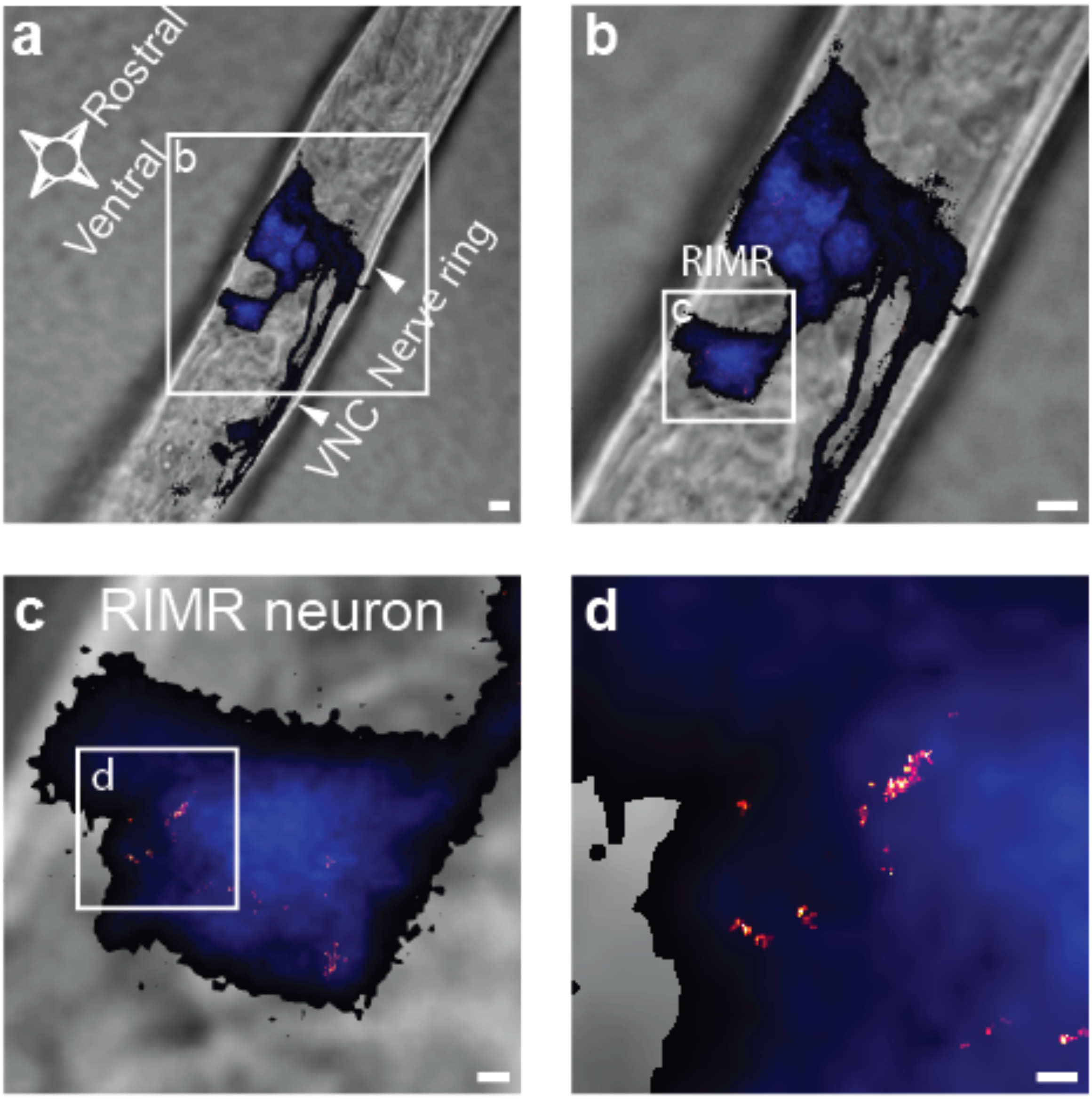

Supplementary figure 7. ccPALM image of RIMR neuron

Partial confocal Z-projection of eGFP (5 optical slices, total thickness of 1.77 μm) fluorescence marking GLR-1 positive neurons and their processes (blue) overlaid with transmission image of the *C. elegans* head region (grey). White arrowheads indicate the nerve ring and the ventral nerve cord (VNC). (**b**) Enlargement of GLR-1 expressing head neurons from Z-projection in (a). Confocal images allow for easy cell identification, e.g. the right RIM (RIMR) neuron cell body is annotated. (**c**) Close up of the RIMR neuron in (b) with the super-resolution distribution of GLR-1 mapped onto the neuron. (**d**) Close up of box in panel (c). The cluster size is not uniform and can be estimated roughly between 50 and 150 nm. Scale bars indicate 2 μm in panels (a) and (b), 250 nm in panel (c) and 100 nm in panel (d). Cut off resolution of all PALM images (c-f) is 20 nm.

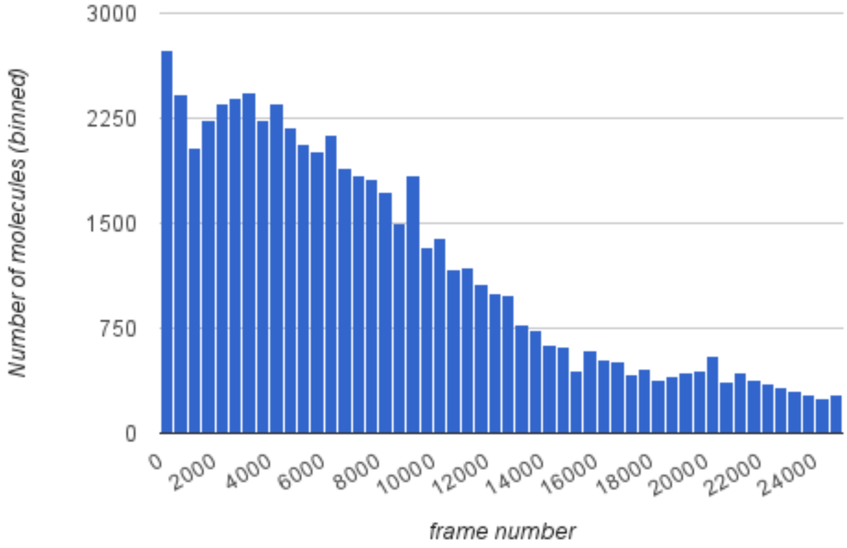

Supplementary figure 8. Detections over time

The graph shows the number of detections over time, expressed as frame number (1 frame equals 50 ms). Over the course of the imaging experiment we increase the 405 nm laser power from approx. 0.5 μW output power to approx. 15 μW output power as to control to amount of photoconverted mEOS2 molecules. The experiment continues to observe all photoconverted mEOS2 molecules as not to undercount the amount of receptors. The operator stops the experiment when virtually no molecules can be observed anymore.

Fw_mEOS2	CCCCCCACCGGTAGAAAAAATGAGTGCGATTAAGCCAGACATG
Rev_mEOS2	CCCCCCGAATTCTTATCGTCTGGCATTGTCAGGC
Fw_glr-1	CCCCCCTCTAGAATCTTCTAAACTTGCTTCTACGG
Rev_glr-1	CAACAACCCGGGCAGCGAGTAACAAATTTAAGAC
Fw_Pglr-1	CCCCCCTCTAGAATCTTCTAAACTTGCTTCTACGG
Rev_Pglr-1	CAACAACCCGGGTGTGAATGTGTCAGATTGGGTGC
Fw_eGFP	CCCCCCACCGGTAGAAAAAATGGTGAGCAAGGGCGAGGAG
Rev_eGFP	CCCCCCGAATTCTTACTTGTACAGCTCGTCCATGCCG

Supplementary Table 1. Primers used for PCR.

References

1 Chang, H. C.-H. & Rongo, C. Cytosolic tail sequences and subunit interactions are critical for synaptic localization of glutamate receptors. *J Cell Sci* **118**, 1945-1956, (2005).
